# The Function and Catalysis of 2-Oxoglutarate-Dependent Oxygenases Involved in Plant Flavonoid Biosynthesis

**DOI:** 10.3390/ijms15011080

**Published:** 2014-01-15

**Authors:** Ai-Xia Cheng, Xiao-Juan Han, Yi-Feng Wu, Hong-Xiang Lou

**Affiliations:** Key Laboratory of Chemical Biology of Natural Products, Ministry of Education, School of Pharmaceutical Sciences, Shandong University, No. 44 West Wenhua Road, Jinan 250012, China; E-Mails: aixiacheng@sdu.edu.cn (A.-X.C.); hanqingmanyu@sina.cn (X.-J.H.); wendywu.12@163.com (Y.-F.W.)

**Keywords:** flavonoid synthesis, 2-ODD oxygenases, flavone synthase I, flavonol synthase, anthocyanidin synthase, flavanone 3β-hydroxylase

## Abstract

Flavonoids are secondary metabolites derived from phenylalanine and acetate metabolism. They fulfil a variety of functions in plants and have health benefits for humans. During the synthesis of the tricyclic flavonoid natural products in plants, oxidative modifications to the central C ring are catalyzed by four of Fe^II^ and 2-oxoglutarate dependent (2-ODD) oxygenases, namely flavone synthase I (FNS I), flavonol synthase (FLS), anthocyanidin synthase (ANS) and flavanone 3β-hydroxylase (FHT). FNS I, FLS and ANS are involved in desaturation of C2–C3 of flavonoids and FHT in hydroxylation of C3. FNS I, which is restricted to the Apiaceae species and in rice, is predicted to have evolved from FHT by duplication. Due to their sequence similarity and substrate specificity, FLS and ANS, which interact with the α surface of the substrate, belong to a group of dioxygenases having a broad substrate specificity, while FNS I and FHT are more selective, and interact with the naringenin β surface. Here, we summarize recent findings regarding the function of the four 2-ODD oxygenases and the relationship between their catalytic activity, their polypeptide sequence and their tertiary structure.

## 1. Introduction

**Figure 1.**
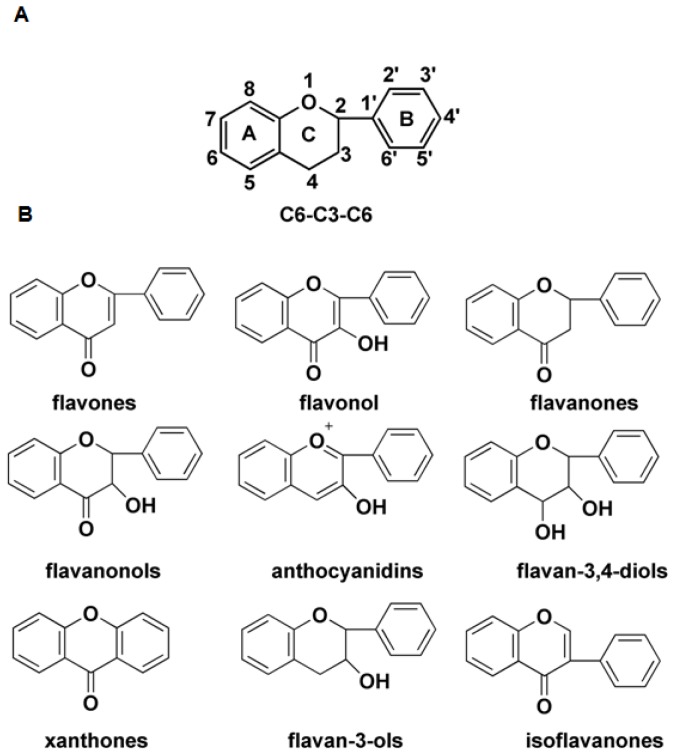
The structure of the flavonoids. (**A**) Flavan (C6–C3–C6) skeleton of flavonoids; (**B**) The oxidation status and saturation of the heterocyclic ring is used to classify the flavonoids.

The flavonoids represent a large group of plant secondary metabolites. To date, some 10,000 different flavonoids have been characterized, compared with about 12,000 alkaloids and about 30,000 terpenoids [1–3]. The interest in this class of compounds reflects their structural diversity, their biological and ecological significance and their health-promoting properties [4]. Their structure comprises a 15 carbon atom phenylpropanoid core, along with two six carbon aromatic rings (denoted as rings A and B) joined by a heterocyclic ring which contains three carbon atoms (ring C), which together can be represented as C6–C3–C6. The core sequence can be extensively modified by rearrangement, alkylation, oxidation and glycosylation [5]. Modifications to ring C differentiate the various flavonoid sub-groups, namely the flavones, isoflavones, dihydroflavonols, flavonols, flavan-3-ols and anthocyanins (Figure 1). Flavonoids are responsible for the non-green pigmentation in both the flower and other parts of the plant, and act as signaling compounds in various plant—microbe and plant—insect interactions [6,7]. In addition to their important physiological roles in plants, flavones have been demonstrated to show significant pharmacological activities, including antioxidant, antiproliferative, antiangiogenic, and neuropharmacological properties of flavonoids [8–10].

**Figure 2.**
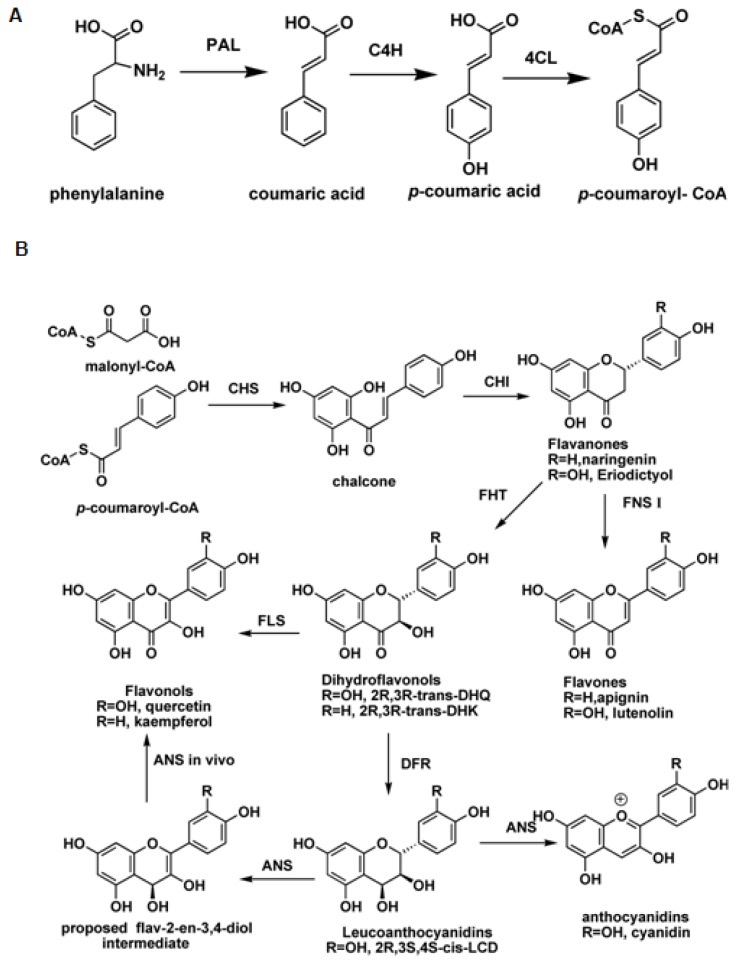
The biosynthesis pathway of flavonoids. (**A**) The biosynthesis of *p*-coumaroyl-CoA; (**B**) The biosynthesis of different kinds of flavonoids from *p*-coumaroyl-CoA and malonyl-CoA. PAL: Phenylalanine ammonia-lyase, C4H: Cinnamic acid 4-hydroxylation, 4CL: 4-Coumarate:Coenzyme A ligase, CHS: Chalcone synthase, CHI: Chalcone isomerase, FHT: Flavanone 3β-hydroxylase, FNS I: Flavone synthase I, FLS: Flavonols synthase, DFR: Dihydroflavonols reductase, ANS: Anthocyanidin synthase.

Due to their importance in genetic studies and their biomedicinal properties, flavonoid biosynthesis is of interest to both biologists and chemists [5]. The biochemistry of flavonoid metabolism has been elucidated over the course of many years through the careful identification and characterization of numerous enzymes. They are generated via the well-characterized phenylpropanoid pathway. Most of the genes encoding the key synthetic enzymes underlying the core structure have been isolated and characterized in various plant species [11]. The first three steps of the pathway involve catalysis by phenylalanine ammonia lyase (PAL), cinnamate 4-hydroxylase (C4H) and 4-coumaroyl CoA ligase (4CL) (Figure 2A). Phenylalanine ammonia lyase catalyzes the deamination of phenylalanine to cinnamic acid, C4H catalyzes the oxidation of cinnamic acid to 4-coumaric acid, and 4CL activates 4-coumaric acid to 4-coumaroyl-CoA. In some plant and bacterial species, tyrosine ammonia lyase (TAL) is utilized for the direct conversion of tyrosine into 4-coumaric acid, bypassing the C4H intermediate. Flavonoid synthesis proceeds from *p*-coumaroyl-CoA and three malonyl-CoA thioesters to chalcone, a reaction enabled by the type III polyketide synthase chalcone synthase; this represents the first committed step towards flavonoid synthesis (Figure 2B). The subsequent ring closure, catalyzed by chalcone isomerase (CHI), produces naringenin, which in turn serves as precursor for a large number of different flavonoids. From naringenin, the pathway diverges into several side branches, each resulting in a different class of flavonoids. Naringenin can be hydroxylated by flavanone 3β-hydroxylase (F3H or FHT) to form dihydroflavonol, which can subsequently be reduced to leucoanthocyanidin, thereby initiating the synthesis of anthocyanin directed by dihydroflanonol-4-reductase and anthocyanidin synthase (ANS, also known as leucoanthocyanidin dioxygenase) [5,12,13]. Alternatively, flavonol synthase (FLS) directs the oxidation of dihydroflavonol to flavonol [14,15]. In the Apiaceae family, flavone synthase I (FNS I) acts to desaturate (2*S*)-flavanones [16–18]. The modification of the central C-ring of flavonoids by these oxygenases and reductases makes the structural diversification of flavonoids. These four oxygenases: flavone synthase I, flavanone 3β-hydroxylase, flavonol synthase and anthocyanidin synthase, known to catalyze modification of the C-ring in plants are Fe^II^ and 2-oxoglutarate dependent oxygenases.

**Figure 3.**
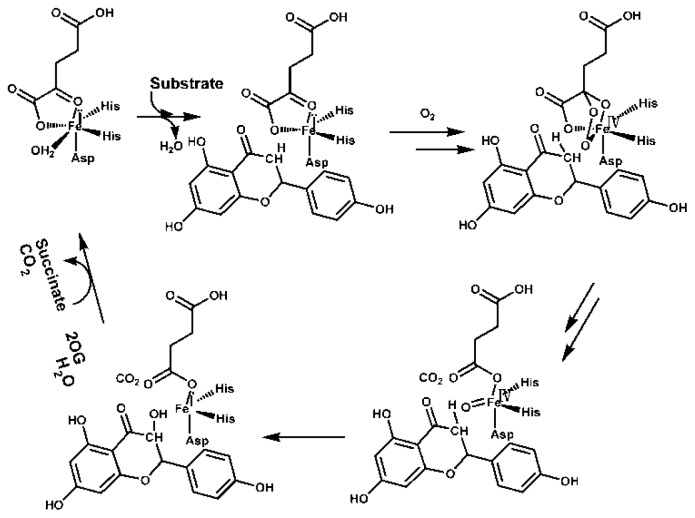
Outline of the proposed catalytic mechanism for 2OG oxygenases. The iron is bound by the HX···H facial triad and the remaining coordination sites are occupied by 2OG and water. Water is displaced upon substrate binding, giving a strongly oxidizing Fe(IV)=O species.

**Figure 4.**
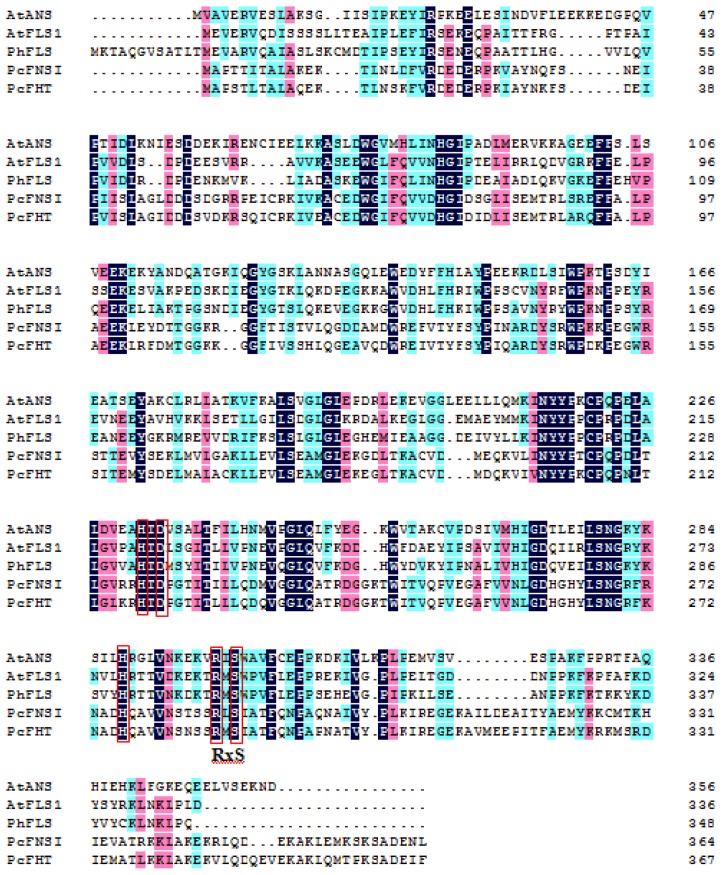
Alignment of the deduced amino acid sequence of different 2-ODDs. Multiple sequence alignment was calculated with the DNAMAN package. Black shading shows amino acid identities. The catalytic residues conserved in these 2-ODDs are indicated in red boxes. The abbreviations for species and accession numbers are: AtANS (*Arabidopsis thaliana* AJ564262), AtFLS1 (*Arabidopsis thaliana* AAB41504), PhFLS (*Petunia hybrida* CAA80264), PcFNS I (*Petroselinum crispum* AY817680), and PcFHT (*Petroselinum crispum* AY230248).

Plant 2-ODD oxygenases constitute a class of non-heme, iron-containing cytosolic enzymes which generally (but not exclusively) require as coreductant 2-oxoglutarate. Their *in vitro* stimulation can often be achieved by the addition of a reducing agent such as ascorbate, which is thought to help to stabilize the Fe^II^ redox state of the enzyme cofactor [19]. The enzymes have been implicated in a variety of plant metabolic pathways, including the synthesis of amino acids, hormones, signaling molecules and a variety of secondary metabolites [20]. They have been shown to be an important component of both hypoxic signaling and DNA repair [21–24]. In the currently proposed consensus mechanism, ferrous iron binds first to the apo-enzyme followed by 2OG (Figure 3). There is evidence, at least in some cases, that binding of iron and 2OG results in parts of the overall structure becoming more ordered [25,26], perhaps to aid in the binding of the “prime” substrate that occurs next [27]. Current evidence suggests that catalysis by 2-ODD oxygenase proceeds via the bidentate binding of the substrate to the Fe^II^ ion present in the enzyme’s active site [28,29]. Substrate binding is thought to enable dioxygen to displace a ligating water molecule from the center of the catalytic Fe^II^ [30,31]. Crystallographic analysis has demonstrated that the *Arabidopsis thaliana* enzyme AtANS contains the characteristic 2-ODD oxygenase double-stranded α helix and has identified which residues are involved in substrate binding [32]. Eight specific residues are perfectly conserved across the whole group, including two histidines (His221, His277; numbers refer to the AtFLS1 sequence), one aspartic acid (Asp223), one arginine (Arg287) and one serine (Ser289). In addition, the motifs Hx(D)xnH and RxS, which are required for binding, respectively, Fe^II^ iron and the substrate (Figure 4) [33,34], are conserved. The Fe^II^ ion is coordinated to the His-rich motif, which is frequently in the form Hx(D)xnH [33]. The four conserved residues Gly68, His75, Gly261 and Pro207 have no clear functionality, but are probably required to ensure correct folding [19]. A further five residues (His132, Phe134, Lys202, Phe293 and Glu295) are less well conserved, but are thought to be determinants of substrate binding [14].

Flavonoid synthesis involves the three common 2-ODD oxygenases FHT (hydroxylating), FLS (desaturating) and ANS (hydroxylating/dehydrating) activities are widely distributed. A fourth-FNS I (desaturating) is confined to Apiaceae species [17,18] and rice [35]. The 6-hydroxylation of the flavonoid molecule catalyzed by a 2-ODD oxygenase has been reported in *Chrysosplenium americanum* [36] and a Cytochrome P450 from soybean [37]. These four common plant 2-ODDs are divided two distinct subgroups, one containing FNS I and FHT and another containing FLS and ANS. FNS I and FHT appear to possess a narrower substrate selectivity than either ANS or FLS.

## 2. Flavonol Synthase and Anthocyanidin Synthase

### 2.1. Flavonol Synthase

FLS (EC number 1.14.11.23) catalyzes the desaturation of (2*R*,3*R*)-*trans*-dihydroflavonols (e.g., dihydroquercetin and dihydrokaempferol) to produce the respective flavonol (quercetin and kaempferol). The enzyme can also mediate the oxidation of naringenin to both dihydrokaempferol enantiomers, an activity normally associated with FHT [38]. FLS activity was first reported in irradiated parsley cells and has been characterized *in vitro* as a soluble 2-ODD oxygenase [39]. Its activity was subsequently detected *Matthiola incana* [40], *Petunia hybrida* [41] and *Dianthus caryophyllus* [42]. The first FLS cDNA to be cloned was from *Petunia hybrida* and was validated by its heterologous expression in yeast. Its insertion as an antisense transgene markedly reduces FLS synthesis in the petals [43]. Subsequently, it has been shown that an *A. thaliana* gene encoding FLS was induced in etiolated seedlings soon after the plants had been exposed to white light [44]. FLS genes have since been characterized from a range of species Arabidopsis [14,15,45,46], *Vitis vinifera* [47], *Camellia sinensis* [48] and *Zea mays* [49]. The FLS peptide sequences are well conserved with one another (about 85% similarity and 50% identity) and also share a significant level of similarity (50%–60%) with ANS; however they are not closely related to those of either FNS or FHT. FLS is a bifunctional enzyme, capable of oxidizing both flavanones and dihydroflavonols. However, the product of all FLS genes characterized so far completely lacks any FNS activity. Like those of other dioxygenases, the FLS sequence also features the two His, one Asp, one Agr and one Ser residues required for Fe^II^ [50] and 2-oxoglutarate [19] binding.

The use of *A. thaliana* as a model for exploring the biochemistry and genetics of secondary metabolism [11] has led to the discovery of at least 35 genes committed to flavonoid synthesis. While most of the enzymes involved in the flavonoid synthesis pathway appear each to be encoded by a single copy gene, the *A. thaliana* genome also contains a small family of six FLS isogenes [44,51]. The product of AtFLS1 (At5g08640) was identified as the enzyme which catalyzed *in vitro* the formation of quercetin and kaempferol from their corresponding dihydroflavonol precursors [38,52]. Although AtFLS1 is mostly important for its influence over tissue flavonoid level [15,45,46], a loss-of-function mutant was still able to accumulate significant quantities of flavonol (whereas an analagous mutant for CHS produced no flavonoids). The implication is that FLS1 is not solely responsible for FLS activity. Each of the deduced products of the five genes AtFLS2 through AtFLS6 has a high degree of similarity to FLS1, and the genes are present as a cluster, suggesting their origin via gene duplication. Nevertheless, a number of motifs present in AtFLS1 and heterologous FLS proteins are not present in any of AtFLS2-AtFLS6. Specifically, these are PxxxIRxxx-EQP at the *N* terminus, CPQ/RPxLAL (upstream of the 2-oxoglutarate binding motif) and SxxTxLVP (downstream of the second conserved region of 2-ODD enzymes). These regions of the protein are potentially important for its FLS activity, and separating FLS from 2-oxoglutarate-Fe^II^-dependent dioxygenases with other substrate specificities. An *N* terminal truncated FLS1 variant (lacking the first 21 residues) showed no *in vitro* FLS activity, which indicated the importance of the PxxxIRxxxEQP motif [15]. Further analysis has shown that the FLS6 sequence features a premature stop codon, and the FLS4 has an additional intron, both of which generate a truncated protein. In the FLS2 sequence, mis-splicing of the second intron induces a frameshift which also results in a truncated protein lacking the key C terminal residues required for substrate and Fe^II^ binding. So FLS2, FLS4 and FLS6 appear to be pseudogenes because of their considerably truncated *C*-termini.

Both FLS3 (At5g63590) and FLS5 (At5g63600) appear to encode full-length proteins and their respective transcript is detectable in seedlings. However, FLS3, but not FLS5, has FLS functionality [46]. It was shown previously that marginal changes in the binding sites of substrate, ferrous iron and oxoglutarate cosubstrate by 2-ODDs may strongly affect the substrate and/or product specificity [53]. Sequence alignment has revealed that in FLS5, an Arg/Lys shift has occurred at position 219 and an Ala/Glu at position 327 [15]. The putative binding sites are fully intact in FLS3. Essential residues in FHT and FNS I have been identified using homology modelling [18]. Models of the LDOX-naringenin [54] and LDOX-dihydroquercetin [53] complexes were therefore employed as templates for modelling the FLS proteins, since the same substrates are involved [38,55]. The analysis identified putative binding sites distributed similarly in the FSL1 and FSL3 polypeptides; those for 2-oxoglutarate (298Arg in LDOX) and Fe^II^ (232His, 288His and 234Asp in LDOX) were similarly distributed in FLS1 and 3 (319Arg, 251His, 309His and 253Asp). A role of 327Glu in substrate binding was also indicated (this residue is absent in FLS5). Although AtFLS3 (At5g63590) has been identified as a second active FLS [46], the remaining flanonol glycosides found in the *fls1-2* mutant was demonstrated to be synthesized in planta by the FLS-like side activity of the LDOX enzyme by using a leucoanthocyanidin dioxygenase (*Idox*) *fls1-2* double mutant [45].

A UV-B-inducible maize FLS has been identified using homology with AtFLS1; this enzyme was capable of converting dihydrokaempferol and dihydroquercetin to, respectively, kaempferol and quercetin. However, unlike AtFLS1, it failed to convert naringenin to dihydrokaempferol. As a transgene, ZmFLS1 was found able to partially complement the flavonol deficiency of the *fls1* mutant and to restore anthocyanin accumulation to wild type levels [49]. A recombinant form of the *Gingko biloba* GbFLS protein was able to catalyze the formation of kaempferol from dihydrokaempferol, consistent with its involvement in flavonol synthesis. However, at least *in vitro*, it also converted naringenin to kaempferol. Thus, based on its sequence and function, this enzyme is a multifunctional dioxygenase which may mediate a range of dioxygenase activity in the flavonoid synthesis pathway [56].

### 2.2. Anthocyanidin Synthase

ANS (alternatively leucoanthocyanidin dioxygenase, EC number 1.14.11.19) is a 2-oxoglutarate and Fe^II^ dependent oxygenase which catalyzes the penultimate step in the synthesis of anthocyanin, converting the colourless leucoanthocyanidin to the pigmented anthocyanidin. ANS exhibits significant similarities in peptide sequence with other 2-ODD oxygenases. Transposon tagging provided the means to isolate the first ANS gene from maize [57], but a detailed biochemical investigation of the enzyme was not mounted until the analysis of *Perilla frutescens* ANS [58]. *In vitro* studies based on ANS from *Petunia hybrida*, *Antirrhinum majus, Zea mays*, and *Torenia fournieri* have confirmed that the enzyme is a member of the 2-ODD dioxygenase family, requiring Fe^II^, 2-oxoglutarate, dioxygen and ascorbate for its activity. These experiments have demonstrated that the enzyme catalyzes the formation of anthocynidin from the natural leucoanthocyanidin substrates via stereo-selective C-3 hydroxylation. *In vitro*, the reaction produces mainly *cis*- and *trans*-dihydroquercetin, along with quercetin, but only traces of the anticipated product cyaniding [59]. Activity has also been demonstrated when the enzyme is presented with trans-dihydroquercetin, a natural substrate for FLS; this reaction generates quercetin, indicating an overlap between the substrate and product selectivities for the 2-ODD oxygenases involved in flavonoid synthesis [60,61]. Both ANS and FLS also react with the non-natural substrate naringenin.

The determination of the crystal structure of anthocynidin synthase is the first of a plant 2-oxoglutarate dependent oxygenase. The crystal structure of ANS has revealed a multicomponent active site containing a metal ion, the co-substrate, and two molecules of the substrate analogue dihydroquercetin [61]. The 2-oxoglutarate binding site is more deeply buried than that of its prime substrate, consistent with the observation that the initial binding of the enzyme with Fe^II^ is followed by binding with 2-oxoglutarate, the substrate and finally dioxygen. Co-crystallization of ANS with (±)-naringenin has shown that the reaction most likely proceeds via hydroxylation cis to the B ring at the C-3 position, as also suggested by the production of cis-dihydrokampferol when ANS is provided with naringenin [61]. The crystal structure has also been used as a template for homology modelling aiming to identify the most likely residues required for FLS, FHT and FNS activity [38,55]. The conserved binding sites for 2-oxoglutarate and Fe^II^ proved to be distributed similarly in ANS and FLS, at least in *A. thaliana*.

## 3. Flavone Synthase I (FNS I) and Flavanone 3β-Hydroxylase (FHT)

Flavanone 3β-hydroxylase (FHT EC Number: 1.14.11.9) and flavone synthase I (FNS I EC Number: 1.14.11.22) share a sequence identity of 80%, even though their biochemical roles are quite distinct. FHT acts early in the flavonoid synthesis pathway, where it catalyzes the hydroxylation of (2*S*) flavanones to (2*R*/3*R*)-dihydroflavonols in plants as a key step towards the biosynthesis of flavonols, anthocyanins and catechins. In contrast, FNS I catalyzes the formation of flavones, such as apigenin, via the 2,3-desaturation of natural flavanones (2*S*)-naringenin. FNS I expression is peculiar to Apiaceae species and one in rice; other plant species which accumulate flavones rely on FNS II, a cytochrome P450-dependent monooxygenase. FHT and either FNS I or FNS II compete for the substrate naringenin. The evolution of FNS I in the Apiaceae suggests that flavones are important for survival and propagation. The high degree of similarity (90% at the polypeptide level) between FNS I and FHT of Apiaceae suggests that FNS I probably evolved from an ancestral FHT gene via duplication [17]. The restriction of FNS I to a single plant family contrasts with the ubiquity of the FHT genes, and implies that FNS I arose much later than FHT. The unique set of 2-ODD oxygenases present in the Apiaceae species makes this family particularly attractive for molecular evolutionary studies. The analysis of several Apiaceae 2-ODD oxygenases has shown that FHT and FNS I are phylogenetically closely related to one another, and adopt a tertiary structure similar to that of ANS [16,17,55]. However, the substrate binding of FHT and FNS I differs from that of ANS and FLS. While ANS and FLS show low substrate specificity and attack the α surface of the substrate, FHT and FNS I have a high substrate specificity and attack the β surface [5,54,55]. Glu-306 is the key residue in AtANS facilitating substrate binding via the formation of a hydrogen bond with the 7-hydroxy moiety of naringenin. Its equivalent position in FHT or FNS I of Parsley is represented by Asn-294 [18], which does not engage in hydrogen bonding. The Phe-144 AtANS residue provides a hydrophobic interaction with the substrate and Tyr-142 is capable of forming hydrogen bonds with the 4-hydroxy moiety of the substrate. These residues are not present in either FHT or FNS I, which instead carry, respectively, Ala-133/Ile-131 and Thr-133/Phe-131. Lys-213 has been proposed to participate in protonation and deprotonation during ANS catalysis [53], and this residue is also not present in either FHT or FNS I.

The significance of *C*-terminal sequence of FHT was also investigated by domain swapping research. Domain swapping experiments carried out on petunia FHT have shown that the truncation of 5, 11, 24 or 29 *C* terminal residues does not abolish the wild type enzyme's specific substrate selectivity, although the longer the truncation, the lower the specificity becomes. A chimeric enzyme, in which the 52 *C* terminal residues have been replaced by the corresponding sequence from *Citrus unshiu* FLS, shows a very low level of FHT activity, but virtually no FLS activity. In contrast to the importance of the *C* terminus with respect to substrate selectivity in the cases of gibberellin C-20 oxidation [62] or deacetoxycephalosporin C synthase from *Streptomyces clavuligerus* [63], the conclusion is that FHT selectivity is not determined by the *C* terminal sequence, which accounts for about 13% of the polypeptide [64]. The variable contribution of the *C* terminus to 2-ODD oxygenase activity has also been demonstrated in parsley FHT and FNS I. Further domain swapping experiments, in which the *N* terminus of parsley FHT has been fused with the *C* terminus of parsley FNS I and vice versa, reveal that the FNS I C terminal portion is also non-essential for its activity. The expression in yeast of two chimeric petunia FHT/FNS I enzymes has shown that in this case, the *C* terminus is important. While the chimera formed from the *N* terminal FNS I 219 residues and the *C* terminal FHT 149 residues is able to convert naringenin to apigenin but not dihydrokaempferol, the one composed from the FNS *C* terminal I 146 residues and the FHT *N* terminal 219 residues shows a weak level of FHT and only a trace of FNS I activity. Truncated forms of FNS I are largely functional [17].

Both FHT and FNS I remove the β-configured hydrogen from C-3 of naringenin, but thereafter FHT catalyzes β-hydroxylation through a rebound process. In contrast, FNS I ensures the syn-elimination of hydrogen from C-2 in a cage-like setting without any intermediate hydroxylation. These subtly different catalytic mechanisms are determined by small polypepdtide sequence differences, involving seven residues at, or close to, the active-site cavity; the key substitutions are M106T, I115T, V116I, I131F, D195E, V200I, L215V and K216R in parsley [18]. Each of Met-106, Ile-115, Val-116, Ile-131, Asp-195, Leu-215 and Lys-216 residues is well conserved across the FHTs. Neither single nor double mutants are capable of transforming FHT into FNS I activity, but a significant amount of FNS I activity can be induced by both of the triple mutations M106T-I131F-D195E and I131F-L215V-K216R, which also retain a reduced level of FHT activity. However, the triple mutant D195E-L215V-K216R shows no FNS I activity, which emphasizes the importance for FNS I activity of Phe-131, even though the single mutant for this residue shows no FNS I activity. Four or five residue substitutions, including I131F, exhibit predominantly FHT activity but also express some FNS I activity. Finally, the replacement of all seven residues induces a near complete change to FNS I activity [18].

## 4. Varied Catalytic Mechanisms

The proposed FNS I reaction clearly differs from those proposed for FLS or ANS. Given that small amounts of dihydrokaempferol and kaempferol are formed, ANS is known to first hydroxylate either the C-3 or the C-2 atom of the substrate and then to direct the anti-periplanar elimination of water [61]. However, FNS I converts neither 2-hydroxynaringenin nor dihydroflavonol to flavone [55,65]. Providing ANS with (2*S*)-naringenin mostly results in C-3 hydroxylation to give cis-dihydrokaempferol as the major product; some trans-dihydrokaempferol and apigenin are also produced, but provision of the non-natural substrate (2*R*)-naringenin generates almost equivalent quantities of dihydrokaempferol and apigenin and only little kaempferol. Labelling studies have demonstrated that some desaturation reactions catalyzed by ANS proceed via an initial C-3 hydroxylation followed by dehydration at the active site. Analysis of the crystal complex has revealed that the 3α hydrogen atom lies closer to the Fe^II^ and is presumably attacked first to release apigenin by syn-elimination [54]. Overall, the precision of naringenin fixation with respect to the Fe^II^ in the active-site pocket of ANS determines whether syn-elimination is preferred over hydroxylation. In the case of the “natural” C-2 stereochemistry of (2*S*)-naringenin, C-3 hydroxylation predominates over desaturation by more than nine fold, probably as a result of the inaccessibility of the C-2 hydrogen atom. In contrast, with the (2*R*)-naringenin substrate, desaturation and C-3 hydroxylation occur to approximately the same extent, probably because the C-3 pro-S and C-2 hydrogen atoms are equally accessible to the reactive oxidizing intermediate. The implication is that the ANS-catalyzed desaturation of (2*R*)-naringenin to form apigenin proceeds with a syn-arrangement of eliminated hydrogen atoms rather than via an oxygenated (gem-diol) flavonoid intermediate. Thus, by utilizing flavonoid substrates with different C-2 stereochemistries, the balance between C-3 hydroxylation or C-2, C-3 desaturation mechanisms can be altered. ANS can catalyze desaturation by either oxygenation or non-oxygenation. In the ANS-Fe^II^-2-oxoglutarate-dihydroquercetin-MES crystal structure, two molecules of dihydroquercetin are complexed to the active site. The dihydroquercetin molecule closest to the Fe^II^ ion is the 2*R*,3*R*-trans stereoisomer; its C-3 hydrogen atom is in a position where it can potentially be oxidized with relatively little movement by a Fe^II^ centered, while the C-2 hydrogen atom lies more distant, pointing away from the Fe^II^ center [53]. This observation is consistent with a mechanism involving oxygenation at the C-3 atom during the ANS-driven desaturation of 2*R*,3*R*-trans-dihydroquercetin. A more direct, either concerted or non-concerted, desaturation process appears to be much less likely. The second dihydroquercetin molecule is present at the active site of the crystal (denoted as the 2*S*,3*S*-trans enantiomer), along with a MES molecule derived from the crystallization buffer.

(±)-Naringenin and (±)-eriodictyol, which have different B-ring hydroxylation profiles, are both non-natural flavonone substrates for ANS. When supplied to ANS, hydroxylated dihydroflavonol products (dihydrokaempferol from naringenin) predominate, with some flavone (apigenin). (±)-Eriodictyol is an inefficient substrate for ANS, with its turnover being about 30% of that generated by (±)-naringenin under standard conditions. The flavone component represents a greater proportion of the overall product in assays with (±)-naringenin than those with (±)-eriodictyol. Thus the balance between desaturation and hydroxylation is in part determined by the hydroxylation profile of the flavonoid B-ring. Extrapolations can be made to both the FNS I and FHT reactions. Both enzymes require as substrate a flavanone molecule having an exposed α, β surface H atom [5,55,66]. By analogy with the model for apigenin formation from (2*R*)-naringenin through ANS-catalyzed syn-elimination [54], the stereo-configuration of the (2*R*)-naringenin C-2 atom can be expected to interfere with FNS I catalysis, so that, as observed, FNS I is incapable of processing (2*R*)-naringenin [67]. Thus, perhaps FNS I and FHT approach (2*S*)-naringenin from the opposite site of the ring plane, which requires a mirror-image orientation of substrate and active-site residues. Two combinations of I131F with M106T/D195E or L215V/K216R have been shown to confer FNS I activity, so these substitutions likely influence product specificity by adjusting the substrate position rather than by actively participating in the reaction mechanism. For hydroxylation by FHT, the C-3 atom needs to lie closer to the Fe^II^ ion than to the C-2 atom, whereas the protons at C-2 and C-3 may lie equidistant from the Fe^II^ ion in FNS I. Overall, the conserved differences in FNS I appear to ensure that the substrate fits into the active-site pocket with maximal proximity of H-2 and βH-3 to the catalytic Fe^II^ ion. The large size Phe-131 residue compared to that of Ile-131 supports this notion. Although the detailed effects of selective amino acid substitutions on the overall structure of parsley FHT are unknown, the experimental data place these residues on the proximal side of the active site which determines FHT and FNS I activity.

## 5. Conclusions

Flavonoids are important secondary metabolites, produced by many plants and are therefore common in the human diet. They are involved in a broad range of physiological functions within the plant, and have been frequently associated with health benefits. The flavonoids share a structure comprised of two six carbon aromatic rings and a heterocyclic three carbon ring containing one oxygen atom, whereas the chalcones lack the heterocyclic ring. The flavonoids are grouped into eight different classes, on the basis of the oxidative status of the heterocyclic ring. The structural diversity of the chalcones and flavonoids is achieved by extensive modifications to the basic structure. During the synthesis of the tricyclic flavonoids, oxidative modification to the heterocyclic ring is catalyzed by Fe^II^ and 2-ODD oxygenases, specifically the three enzymes FNS I, FLS and ANS. In contrast, FHT catalyzes hydroxylation at the C-3 pro-R position of (2*S*)-naringenin. ANS/FLS and FHT/FNS form pairs of flavonoid oxygenases, distinct both in terms of their preferred substrate and stereo-selectivity. ANS/FLS are closely related to one another by sequence and only rather distantly to FHT. ANS/FLS and FHT/FNS pairs may be classified as, respectively, α and β surface-selective oxygenases.
